# Short-Term Effect of Standard Automated Perimetry Testing on Intraocular Pressure in Patients with Open-Angle Glaucoma

**DOI:** 10.1155/2013/956504

**Published:** 2013-02-14

**Authors:** Chang Mok Lee, Young Cheol Yoo

**Affiliations:** Department of Ophthalmology, Kangdong Sacred Heart Hospital, Hallym University College of Medicine, 150 Seongnae-gil, Gangdong-gu, Seoul 134-701, Republic of Korea

## Abstract

*Purpose.* To evaluate the short-term effect of standard automated perimetry (SAP) testing on intraocular pressure (IOP) in patients with open-angle glaucoma (OAG). *Methods.* We tested 45 patients (71 eyes) with OAG that had stable IOP under medical treatment. IOP was measured four times using an iCare rebound tonometer (RBT) immediately before, immediately after, 10 minutes after, and 20 minutes after SAP testing. Logistic regression analyses were performed to determine the relationships among SAP test duration, mean deviation of the SAP result, type of glaucoma medications, patient age, and significant IOP change (exceeding 2 mmHg) from baseline IOP. *Results.* The mean baseline IOP was 13.29 ± 3.06 mmHg. Although IOP changes immediately and 20 minutes after SAP testing were not statistically significant, the IOP change 10 minutes after SAP testing (−0.57 ± 1.84 mmHg) was statistically significant. However, the changes were within the margin of error of the RBT. Test duration, mean deviation, patient age, and type of glaucoma medications did not have a significant influence on IOP change (all *P* > 0.14). *Conclusions.* IOP measured by RBT did not vary significantly after SAP testing in patients with OAG. It may be not necessary to reject IOP measured after SAP testing in patients with OAG.

## 1. Introduction

Intraocular pressure (IOP) remains the only treatable risk factor for the management of glaucoma. Repeated IOP measurement and standard automated perimetry (SAP) testing are simple but fundamental procedures used to assess the stage of progression and to determine adequate treatment for patients with glaucoma [1, 2]. On a daily basis in our practice, IOP is measured after SAP testing. Afterward, both the SAP results and IOP are discussed with the patients, and their treatment strategies for glaucoma may be adjusted. There is a possibility that visual field examination performed before IOP measurement using both topical anesthetics and fluorescein dye affects the IOP values, misleading the clinician to strengthen the patients' glaucoma treatment plan.

Currently, there is no consensus in the literature about the effects of SAP testing on IOP. One prospective study reported that IOP varied significantly and tended to increase immediately after SAP testing in patients with primary open-angle glaucoma (POAG), but other studies have reported no significant difference [3–5]. But, there was no trial that evaluates serial changes of IOP values through timeline after SAP testing. The aim of the present study was to evaluate the short-term effects of SAP testing on IOP measurement in patients with open angle glaucoma (OAG). To minimize measurement errors and adverse effects caused by topical anesthesia and repeated corneal applanation by Goldmann applanation tonometry (GAT), we designed a prospective study using the iCare rebound tonometry (RBT) which does not require anesthesia or corneal applanation to measure IOP. To determine if there is a specific time after SAP testing at which IOP increases, we measured IOP immediately after, 10 minutes after, and 20 minutes after SAP testing.

## 2. Methods

We consecutively recruited patients from the glaucoma clinic of Kangdong Sacred Heart Hospital (Seoul, Korea) from August 2011 to January 2012. This study adhered to the tenets of the Declaration of Helsinki and was approved by the Institutional Review Board of the hospital. A pilot study was performed to determine an appropriate sample size for our study. The minimum number of subjects calculated by G*power (G*power 3.1.3; Franz Faul, Uni Kiel, Germany) was 51 (*α* = 0.05, power = 0.8, effect size = 0.48). Selection criteria were as follows: OAG and stable IOP as assessed by GAT at least three different times during a six-month interval before SAP testing. All patients were receiving topical glaucoma medications and were familiar with the SAP test procedures. Exclusion criteria were as follows: nonadherence with glaucoma medical treatments, documented difficulty in IOP measurements, and history of previous ocular trauma or surgery.

All patients underwent a standard procedure with the same order of examinations. IOP was measured at four different times for each subject using the iCare rebound tonometer (Tiolat Oy, Helsinki, Finland). IOP was taken immediately before, immediately after, 10 minutes after, and 20 minutes after SAP testing. At each time point, 6 consecutive measurements were taken and the preprogrammed software determined the average IOP value after automatically discarding the highest and the lowest values of the 6 readings taken in each eye. Only measurements that show no error bar that indicates high standard deviation (which is based on the manufacturer's designation) were included. To minimize the bias of tonometry itself, the same operator (LCM) and the same device were always used. The SAP testing was performed with a Humphrey visual field analyzer II model 750 (Carl Zeiss Meditec, Dublin, CA, USA). Central 24-2 Swedish interactive thresholding algorithm (SITA) standard strategy was used after mesopic adaptation with undilated eyes. During SAP testing, the near prescription lens was set as needed, and the fellow eye was patched. The date and time of SAT testing, SAP test duration, and mean deviation (MD) of SAP results were recorded.

All statistical tests were performed using SPSS (SPSS statistics 19 doctor's pack; Chicago, IL, USA). IOP measured immediately after, 10 minutes after, and 20 minutes after SAP testing were compared with the baseline IOP before SAP testing using two-tailed paired *t*-tests. Subanalyses were performed to evaluate the impact of patient age, SAP test duration, MD, and particular topical glaucoma medications on IOP change after SAP testing. The glaucoma medications were divided into two groups: drugs including *α*2-agonist (AA) or *β*-blockers (BB) or drugs including prostaglandin analogue (PGA) or carbonic anhydrase inhibitor (CAI). AA and BB were classified as the same group, as they are known to be mediators of the sympathetic response with identical intracellular mechanisms that result in reduced activity of membrane-bound adenylyl cyclase [6, 7]. PGA increases uveoscleral outflow by activation of a molecular transduction cascade and an increase in the biosynthesis of certain metallopreteinases [8]. CAI decreases the production of aqueous humor by inhibiting the CA-II enzyme [9]. To determine the relationships of patient age, SAP test duration, MD, and particular glaucoma medications with IOP change greater than 2 mmHg from baseline IOP, logistic regression analyses were performed.

Separately, we consecutively measured the IOPs of 33 healthy subjects (66 eyes) three times using the same RBT as in our previous report [10]. The reproducibility of our RBT equipment was established by calculating intraclass correlation coefficients (ICC).

## 3. Results

Forty-five patients (71 eyes) were included based on the predetermined criteria. Their ages ranged from 42 to 75 years (mean ± SD; 57.4 ± 11.3 years). Twenty-three patients were male and 22 patients were female, with an average age of 57.4 ± 11.4 years and 56.7 ± 11.3 years, respectively. Most of the patients were diagnosed with normal tension glaucoma. Only four patients had primary open angle glaucoma. IOP measured by GAT at previous visits ranged from 8 to 18 mmHg (mean ± SD; 13.21 ± 2.51 mmHg). SAP test duration ranged from 4 : 09 to 11 : 14 minutes (mean ± SD; 5 : 51 ± 1 : 19 minutes). MD ranged from +1.07 to −15.42 dB (mean ± SD; −3.90 ± 8.01). There was no preferential time for SAP testing because the glaucoma clinic appointments were equally distributed between morning and afternoon. All patients were medically controlled with an average of 1.2 glaucoma medications. Thirty-four eyes were being treated with either an AA or BB, while 37 eyes were being treated with either a PGA or CAI only (Table 1).

**Figure 1:**
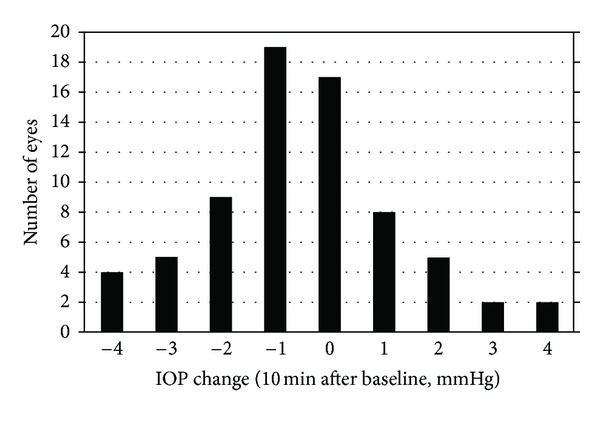
Distribution of intraocular pressure changes found in each subjects 10 minutes after standard automated perimetry testing.

The mean baseline IOP before SAP testing was 13.29 ± 3.06 mmHg. Although IOP changes immediately (−0.21 ± 1.98 mmHg) and 20 minutes (−0.37 ± 2.08 mmHg) after SAP testing were not statistically significant, IOP changes 10 minutes after SAP testing (−0.57 ± 1.84 mmHg) were statistically significant (Table 2). However, the IOP changes found in each subject were very small. Fifty-eight (81.7%) of the 71 eyes showed an IOP change smaller than 2 mmHg. Only 13 eyes (18.3%) showed an IOP change exceeding 2 mmHg.  Figure 1 shows the distribution of IOP changes found in each subject 10 minutes after SAP testing.

The data of IOP change 10 minutes after SAP testing was also analyzed with respect to patient age, SAP test duration, MD, and particular glaucoma medications. Analyses showed that no factor had a significant influence on the IOP changes (exceeding 2 mmHg) measured after SAP testing (all *P* values > 0.14) (Table 3). The ICC of the RBT was established by measuring the IOPs of 33 healthy subjects (66 eyes). The ICC was 0.951 (95% confidence interval; 0.925 to 0.971), suggesting a high degree of reproducibility in our RBT equipment. The variability of the individual measurements was 1-2 mmHg; only 5 eyes (7.6%) among 66 eyes showed differences exceeding 3 mmHg.

## 4. Discussion

Twenty percent to 30% lowering of IOP may be regarded as a treatment goal to prevent the progression or development of glaucoma [11, 12]. Conversely, an overestimated IOP may lead physicians to reinforce treatment plans for glaucoma. In clinical settings, a question often arises regarding the most appropriate time to measure the IOP if SAP testing is scheduled. The reason for measuring the IOP before SAP testing is that most testing except SAP testing is performed in the same room. The SAP testing takes place in another room at the clinic [5]. In addition, some patients experience slightly blurred vision after GAT measurements; the quality of the SAP results might potentially be reduced by a tear film disruption and decreased blinking caused by topical anesthetics and fluorescein dye. Therefore, many clinicians prefer to measure the IOP after SAP testing.

Previous studies have examined the effects of SAP testing on IOP but have shown contradictory results. Recupero et al. [3] first reported that transient IOP increases after SAP testing in most eyes with POAG but not in healthy eyes. In their study, the mean IOP increase was 2.38 mmHg, and IOP returned to the preexamination value after one hour. They believed it would be wise to reject tonometric findings measured soon after SAP testing. Ni et al. [13] conducted a retrospective study that reported a 10.6% increase of the IOP in patients with POAG after SAP testing from the previous visit that reversed upon the subsequent visit. However, in their study, no IOP values were measured immediately before SAP testing. Therefore, it is difficult to say that the IOP increase observed in their study was truly caused by SAP testing. They also did not determine the duration of IOP elevation. On the other hand, two other studies failed to show IOP variance after SAP testing [4, 5]. In these studies, the subject numbers were small, 27 and 21, respectively. The hypothesis that SAP testing increases IOP requires a more detailed prospective study. We wanted to further clarify the true effect of SAP testing on IOP.

In our prospective study, the only statistically significant changes in mean IOP occurred 10 minutes after SAP testing. However, the mean IOP change was very small (−0.57 mmHg) and may be within a clinically acceptable margin of error [14–16]. Moreover, the IOP changes found in each subject were unequal. In some cases, the IOP decreased, and in other cases, it increased. Most of the IOP changes found in each subject were within 2-3 mmHg and well within the previously described normal variability of 2-3 mmHg between examinations when using the RBT [17, 18]. Differences less than 2 mmHg for one examination to another in a given patient can be considered normal variation of the patient and/or the instrument used [19]. To evaluate the factors affecting significant IOP changes (exceeding 2 mmHg), subgroup analyses were performed with respect to patient age, SAP test duration, MD, and particular glaucoma medications. None of these factors were shown to have a significant influence on IOP change.

Recupero et al. [3] hypothesized that decreased accommodation with age may be a factor that reduces aqueous outflow. The influence of accommodation on IOP has been widely studied. Particularly, it has been stated that sustained accommodation causes a decrease in IOP [20]. However, in our study, the patient used an age-related correction for near vision in addition to their own distance refraction, which leaves some room for active accommodation. Another reason for the discrepancy could be age differences. Recupero et al. [3] reported a much higher mean IOP increase after SAP testing in elderly patients (mean age = 69.9). The mean age of the patients in our study was only 57.4 years, which means more capability for accommodation.

Another hypothesis of IOP increase after SAP testing is that SAP is perceived as psychic stress by some patients, leading to a sympathetic response that transiently elevates IOP [21, 22]. There is some evidence in the literature to suggest that environmental stressors may be associated with increased IOP in POAG patients [23]. A longer SAP test duration (central 30-2 full-threshold strategy) is associated with increased psychological stress in the Recupero et al. sample, which could explain the differences with our study (central 24-2 SITA standard strategy). The SAP test duration of our study was short and had no influence on significant IOP change. Furthermore, glaucoma eye drops that do not mediate a sympathetic response had no influence on significant IOP change.

To the best of our knowledge, this study was the first and largest report using an RBT among the previous studies that evaluated the effect of SAP testing on IOP. We used an RBT to minimize measurement errors and adverse effects on SAP results because it does not require corneal applanation or topical anesthesia. To determine the timeline of IOP change caused by SAP testing, we measured the IOP immediately before testing, immediately after testing, 10 minutes after testing, and 20 minutes after testing.

There are some limitations in our study. First, no IOP values were obtained using GAT, which has been considered the gold standard in IOP measurement [24, 25]. However, numerous recent studies have compared RBT with GAT and reported a good agreement between the two devices [26–29]. Some investigators have found that the RBT tended to overestimate IOP with respect to GAT as IOP increased beyond the normal range [30, 31]. In our study, all patients had a stable IOP within the normal range (from 9 to 18 mmHg). In addition, the ICC value calculated in our study was very high (0.951), suggesting a high degree of reproducibility of our RBT equipment [10, 32]. Therefore, IOP values in our study may be slightly different from the actual IOPs of the patients but are assumed to be sufficiently accurate and reliable. Secondly, there was no appropriate control group in our study. IOP is a dynamic function and fluctuates in both normal subjects and glaucoma patients although there is generally more fluctuation in glaucoma patients. Consequently, it is difficult to distinguish IOP change caused by SAP testing from physiologic IOP change during timeline. All IOP measurements in a single subject were performed within 40 minutes in our study. The physiologic IOP variations were thought to be minimal and negligible. Finally, subjects in our study were limited to patients with OAG. Various subgroup analyses could not be performed due to the small number of subgroup subjects. Further study is needed to examine the impacts of angle closure glaucoma and glaucoma operation with respect to IOP change after SAP.

This is the first and largest paper to evaluate the effect of SAP testing on IOP using an RBT. In our study, most of the IOP changes after SAP test found in each subject were within 2-3 mmHg and within the clinically acceptable margin of error. In conclusion, it is not necessary to reject the IOP values measured after SAP testing in patients with OAG under medical treatment.

## Figures and Tables

**Table 1:** Patient characteristics.

	71 eyes of 45 patients
Age (years, mean ± SD)	57.4 ± 11.3
Sex (male : female)	23 : 22
IOP* (mmHg, mean ± SD)	13.21 ± 2.51
SAP test duration (min : sec, mean ± SD)	5 : 51 ± 1 : 19
Mean deviation (dB, mean ± SD)	−3.90 ± 8.01
Number of eyes using glaucoma medication	
Including *α*2-agonist or *β*-blockers	34
Including PGA or CAI only	37

*Measured by Goldmann applanation tonometry at previous visit.

IOP: intraocular pressure; SAP: standard automated perimetry; PGA: prostaglandin analogue; CAI: carbonic anhydrase inhibitor.

**Table 2:** Intraocular pressure measurements before and after standard automated perimetry testing.

	IOP (mean ± SD, mmHg)	IOP change from baseline (mean ± SD, mmHg)	P*
Baseline	13.29 ± 3.06	—	—
Immediately after SAP	13.08 ± 2.91	−0.21 ± 1.98	0.38
10 minutes after SAP	12.71 ± 3.15	−0.57 ± 1.84	0.01
20 minutes after SAP	12.91 ± 3.29	−0.37 ± 2.08	0.14

*Paired *t*-test.

IOP: intraocular pressure; SAP: standard automated perimetry.

**Table 3:** Factors affecting the intraocular pressure changes exceeding 2 mmHg after standard automated perimetry testing.

Factors	Odds ratio (95% confidence interval)	P*
Patient's age	0.958 (0.906–1.011)	0.12
SAP test duration	0.721 (0.348–1.494)	0.37
Mean deviation	0.979 (0.891–1.076)	0.65
Glaucoma drugs		
Including *α*2-agonist or *β*-blockers	0.914 (0.166–4.989)	0.91
Including PGA or CAI only	0.717 (0.129–3.999)	0.70

*Logistic regression analysis.

SAP: standard automated perimetry; PGA: prostaglandin analogue; CAI: carbonic anhydrase inhibitor.
